# Young Men’s Views Toward the Barriers and Facilitators of Internet-Based Chlamydia Trachomatis Screening: Qualitative Study

**DOI:** 10.2196/jmir.2628

**Published:** 2013-12-03

**Authors:** Karen Lorimer, Lisa McDaid

**Affiliations:** ^1^Institute for Applied Health ResearchGlasgow Caledonian UniversityGlasgowUnited Kingdom; ^2^MRC/CSO Social and Public Health Sciences UnitUniversity of GlasgowGlasgowUnited Kingdom

**Keywords:** qualitative research, adolescent male, socioeconomic status, chlamydia, sexually transmitted diseases, screening, Internet

## Abstract

**Background:**

There is a growing number of Internet-based approaches that offer young people screening for sexually transmitted infections.

**Objective:**

This paper explores young men’s views towards the barriers and facilitators of implementing an Internet-based screening approach. The study sought to consider ways in which the proposed intervention would reach and engage men across ages and socioeconomic backgrounds.

**Methods:**

This qualitative study included 15 focus groups with 60 heterosexual young men (aged 16-24 years) across central Scotland, drawn across age and socioeconomic backgrounds. Focus groups began by obtaining postcode data to allocate participants to a high/low deprivation category. Focus group discussions involved exploration of men’s knowledge of chlamydia, use of technology, and views toward Internet-based screening. Men were shown sample screening invitation letters, test kits, and existing screening websites to facilitate discussions. Transcripts from audio recordings were analyzed with "Framework Analysis".

**Results:**

Men’s Internet and technology use was heterogeneous in terms of individual practices, with greater use among older men (aged 20-24 years) than teenagers and some deprivation-related differences in use. We detail three themes related to barriers to successful implementation: acceptability, confidentiality and privacy concerns, and language, style, and content. These themes identify ways Internet-based screening approaches may fail to engage some men, such as by raising anxiety and failing to convey confidentiality. Men wanted screening websites to frame screening as a serious issue, rather than using humorous images and text. Participants were encouraged to reach a consensus within their groups on their broad design and style preferences for a screening website; this led to a set of common preferences that they believed were likely to engage men across age and deprivation groups and lead to greater screening uptake.

**Conclusions:**

The Internet provides opportunities for re-evaluating how we deliver sexual health promotion and engage young men in screening. Interventions using such technology should focus on uptake by age and socioeconomic background. Young people should be engaged as coproducers of intervention materials and websites to ensure messages and content are framed appropriately within a fast-changing environment. Doing so may go some way to addressing the overall lower levels of testing and screening among men compared with women.

## Introduction

Chlamydia trachomatis is a common bacterial sexually transmitted infection (STI) in the United States, United Kingdom (UK), and other European countries [1-3] and disproportionately affects young people under 25 years of age. Chlamydia has been referred to as a “silent epidemic” or “silent infection” due to its largely asymptomatic course [4,5], which provides young people with no or few visible cues with which to seek health care. Chlamydia can be identified via screening (“members of a defined population, who may not know they are at risk of a disease or its complications, are asked a question or offered a test to identify those who are more likely to be helped than harmed by further tests or treatment”) [6]. Screening can be *opportunistic* (“a health professional offers a screening test to patients attending health care or other defined settings for unrelated reasons; the onus is on the health professional to repeat the test offer at appropriate intervals”) or *proactive* (“population registers are used to invite members of the population at risk for screening at appropriate intervals”) [6]. A screening program is one in which there is systematic and organized provision of regular chlamydia testing to reach a defined population—whether by opportunistic or proactive approach [2]. A survey by the European Centre for Disease Prevention Control (ECDC) in 2008 reported no organized chlamydia screening activity across almost half of the countries surveyed [7]; however, nine countries, including France, Ireland, and the Netherlands, had plans to introduce screening programs in the future. More recently, Low et al (2012) conducted a cross-sectional survey of 33 European countries to assess current and planned chlamydia control activities [8]; they also identified nine countries with plans to introduce a screening program (Bulgaria, Finland, Greece, Luxembourg, Slovenia, Turkey, Norway, France, Netherlands), with Norway being the only country exploring and planning a proactive screening approach. An opportunistic screening program has been operating in England since 2007, but as health is devolved within the United Kingdom this does not extend to Scotland, Wales, or Northern Ireland, which have no program.

A number of studies have explored the feasibility and acceptability of home-based sampling, based on uptake, since the introduction of urine-based testing around 2000 [9]. Collectively, these studies indicate that such screening approaches are acceptable and feasible, across a range of populations, settings, and methods (for example, direct mailing of test kits or requests made online) [10]. Studies of home collection of urogenital specimens for direct mailing to a laboratory for testing have been facilitated via websites such as “I Want The Kit” [11] and have found such a method to be acceptable to men. While many early studies focused on women, more recent work has provided evidence on men’s responses to screening invitations and moved the evidence-base toward a more nuanced understanding of acceptability beyond simple uptake rates. For example, an ongoing trial in the Netherlands examining Internet-based screening assessed acceptability via questionnaires [12] and found nonresponse to the screening offer was not due to a lack of Internet access, but was largely based on perceptions of individual risk.

Technology, such as websites, mobile (cell) phones, and short messaging services (SMS or “texts”), offers exciting opportunities for non-clinical approaches to offer convenient, easy, and confidential services, which fit with what young people report they prefer [13]. Smartphone ownership—phones based on an operating system such as Android, Blackberry, or iOS, with Web access, “apps”, and ability to synchronize email—is increasing, particularly over recent years and among younger people [14]. This increases opportunities to deliver a service straight into young people’s pockets and for interventions to be available when young people demand them. The feasibility and acceptability of different forms of technology in sexual health promotion are being explored, with emerging evidence of increased acceptability and effectiveness [15-19]. Recent service-focused findings demonstrate technology facilitates improvements in partner notification, access to diagnostic tests such as chlamydia screening, appointment keeping, and notification of medical investigations [18,20-25]. The neighboring literature on HIV prevention suggests information and communication technology (ICT) has the capacity for broad diffusion of prevention activities as well as targeting and tailoring of services and messages [21,26]. A particular strength is the ability of ICT to enable a shift toward routine testing, with reduced time burdens and costs [21].

Continued exploration and evaluation of these screening approaches should take account of not just the reach of screening—in terms of overall numbers of people screened as a proportion of those eligible for screening—but also the variation in testing by, for example, age, gender, and class, or combinations in terms of intersectionality. This kind of monitoring will contribute to a greater understanding of inequalities in screening uptake [27]. Currently, men are most often included at the periphery of chlamydia screening approaches and programs, mostly via partner notification. Even efforts to target men alongside women have resulted in more women being screened than men, which results in missed opportunities for men and also missed opportunities for primary prevention for women—as screening men reduces the prevalent reservoir of infection from which women acquire chlamydia [28]. Economic modelling, which explored the options of men being included as primary screening cases or via partner notification, found the former would lead to a faster and greater reduction in overall prevalence [29]. Sheringham et al (2011) explored social variations in the delivery of the National Chlamydia Screening Programme (NCSP) in England to assess whether screening was reaching those in deprived areas of England [30]. They found screening coverage was highest in more socioeconomically deprived areas where chlamydia positivity was also highest. However, Woodhall et al (2012), who also analyzed data from the NCSP in England to describe who was using an Internet-based screening approach, found Internet-based testing was more evenly distributed across areas of high deprivation than either clinic or community-based approaches within the NCSP [31]. It may be that the Internet has driven, or at least in some way facilitated, a greater uptake among young people in deprived areas.

Although somewhat limited, there is growing evidence of what encourages young people to engage with a screening offer in the first place. Internet-based interventions, such as that by Kang and colleagues [32], suggest the method of contacting young people (eg, email) may have an impact on engaging them. Other work, such as “Sexunzipped”, provides a window into what young people want from a general sexual health website [33]. In the Sexunzipped study, the focus group data with 67 young people aged 16-22 years found a desire for straightforward information, a degree of interactivity with peers online, and to see themselves in images or video material online [33,34]. This study is a good example of involving young people in shaping the design of online sexual health interventions; nevertheless, we have a paucity of data that involves young people themselves as co-producers of interventions and assesses whether this affects the effectiveness of chlamydia screening efforts. It may be challenging to take the time to seek young people’s views and integrate them into intervention designs within a fast-paced environment of shifting technology, but such challenges should be faced if it leads to a highly acceptable sexual health intervention for young people.

The intent of this study was to gather evidence to inform the subsequent design of an Internet-based approach to chlamydia screening targeting young men (aged 16-24 years). To aid the development of our intervention, we aimed to explore the barriers and facilitators to implementing an Internet-based chlamydia screening approach, including the acceptability of such an approach. We sought to explore differences in the views of young men, by sample characteristics including age group (16-19 years and 20-24 years) and deprivation.

## Methods

### Design and Setting

Participants were selected purposely to include a range of characteristics, including age, level of deprivation in their area of residence, ethnicity, residing in an urban or semi-rural area, and current employment status (unemployed, in school, or employed). We aimed to recruit an even number of groups by age group (split into two groups: 16-19 years and 20-24 years). We also made a deliberate effort to recruit men from areas of high deprivation, using the Scottish Index of Multiple Deprivation (SIMD) to identify areas of high and low deprivation across central Scotland. The SIMD is a measure of relative deprivation, derived from the ranking of small areas (using postcode [zip code] data) as most deprived (1) through to least deprived (5). The Scottish Government website provides an interactive map to identify the SIMD rank of small areas [35]. The 2012 SIMD combines 38 indicators across 7 domains, namely: income, employment, health, education, skills and training, housing, geographic access, and crime; the overall index is a weighted sum of the 7 domain scores. Our purposive strategy to select young men allowed us to explore within our data whether there were differences in the views of men by their characteristics, in particular by age and deprivation. We sought the postcode (zip code) of each participant at the interview to check they were from the SIMD area we were recruiting within and to enable us to categorize groups as being from areas of high or low deprivation.

### Recruitment

Men were recruited via a range of non-clinical settings, including workplaces, health and fitness settings, community groups, and further education settings (post-high school age but lower than university level). A mixture of purposive and snowball sampling was used to ensure a heterogeneous sample for a range of characteristics: age, socioeconomic background, and ethnicity. Focus groups were homogenous by age group, ethnicity, and deprivation.

### Data Collection

**Figure 1:**
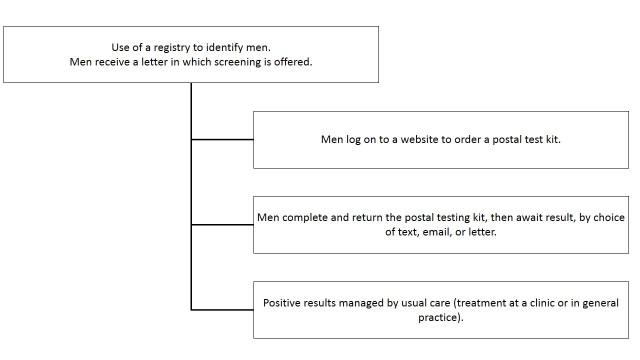
Process of Internet-based proactive screening provided to the young men.

Focus groups lasted between 1-2 hours and took place in private spaces made available by our community partners or at the university, with the same facilitator. At the start of each focus group, after consent forms were completed, participants were asked to verbally confirm their postcode. The ensuing focus group discussions focused on knowledge of chlamydia, technology use and attitudes towards smartphones and the Internet, and views on sample screening letters and websites. Focus groups began with participants being asked to describe their knowledge of chlamydia and then technology use, including use of a mobile (cell) phone and the Internet. Participants were invited to reflect on the amount of access they had to, and their use of, such technologies, how private their use was, and their desire for more or less technology use. Insights were then gained from men about their willingness to participate in a proactive screening approach, which made use of the Internet and postal testing kits. To facilitate these discussions, we described the proposed proactive approach to screening as shown in Figure 1.

Young men were first shown three sample screening invitation letters (each were different in order to elicit their style and content preferences) to be sent from GPs (general practitioner), or via a central register, and then a sample postal test kit, before being shown on a laptop existing UK-based websites offering chlamydia screening. Five sites were shown, with each chosen to present a range of styles and content for the men to comment on their preferences (see Multimedia Appendix 1). A semi-structured topic guide was designed to guide participants through these topic areas in order to build a picture of potential barriers and facilitators to a proactive, Internet-based approach to chlamydia screening.

### Data Analysis

Group discussions were audio-recorded and transcribed verbatim and checked. QSR NVivo 10 was used to facilitate analysis. Transcripts were read repeatedly by the researcher and a thematic coding framework was developed as a collaborative effort within the research team (including KL and LM); we then used the “Framework” approach, where data are coded, indexed, and charted systematically, then organized using a matrix or framework [36]. The five key stages of Framework are: familiarization, identifying a thematic framework, indexing, charting, mapping, and interpretation. Framework analysis begins deductively from the study aims and objectives (generating prepositions), but is also inductive (using patterns and associations derived from observations) [37]. Constant comparison was carried out to check for deviant cases as well as similarities, in an iterative process. During analysis, we explored participants’ attributes (eg, age, deprivation), in which we had an a priori interest, against the various themes to rigorously explore emergent patterns in response, particularly by age and deprivation.

### Ethics

Ethical approval was obtained from Glasgow Caledonian University School of Health and Life Sciences Ethics Committee. Participants provided written informed consent and permission for the discussion to be audio-recorded and received a £10 (US$16) payment for their time, in the form of a voucher, which they could spend in a variety of shops.

Illustrative quotes are used throughout indicating the focus group, age, and deprivation category of the respondents.

## Results

### Participant Characteristics

Fifteen focus groups were conducted with men aged 16-24 years (n=60 individuals), with a minimum of 3 and maximum of 5 participants in the groups. The young men were sociodemographically diverse and most groups consisted of pre-existing friendship or work networks. In only one group did the participants not know each other. Table 1 shows demographic information about the groups. Of the 15 groups, 8 were of men aged 16-19 years and 7 with men aged 20-24 years. Nine groups were of men from deprived areas and 6 from non-deprived areas. Most (11/15) were from urban areas.

**Table 1:** Focus group participants (groups n=15; individuals n=60).

Focus group	Aged 16-19 years	Aged 20-24 years	White British	BME^a^	Deprived (SIMD^b^ 1 or 2)	Non-deprived (SIMD 4 or 5)	Urban	Semi-rural
1	✓		✓		✓			✓
2	✓		✓		✓			✓
3	✓		✓		✓			✓
4	✓		✓		✓			✓
5	✓		✓			✓	✓	
6	✓		✓			✓	✓	
7		✓	✓			✓	✓	
8		✓	✓			✓	✓	
9	✓		✓			✓	✓	
10	✓		✓		✓		✓	
11		✓	✓		✓		✓	
12		✓		✓	✓		✓	
13		✓		✓		✓	✓	
14		✓	✓		✓		✓	
15		✓	✓		✓		✓	
Total	8	7	13	2	9	6	11	4

^a^BME: Black and Minority Ethnic

^b^SIMD: Scottish Index of Multiple Deprivation

### Men’s Technology Use

Young men were invited to describe their use of technology, particularly their phone and Internet usage. While most men used the Internet every day, their use was heterogeneous in terms of individual practices using new technologies. All participants used a mobile phone and most phones were Internet-enabled (eg, Blackberry device or iPhone), with only two men having a phone with no Internet function, but with Internet access elsewhere. Most men described “being online” for a few hours each day, including frequent Internet browsing on their phone (checking Facebook, email, sports, and news websites), as well as computer-based game play and browsing to socialize. Some described infrequent Internet usage, whether on a phone or computer, for activities such as checking email and browsing websites. Nevertheless, almost every man reported using Facebook on a daily basis.

While there were some differences in Internet use between groups from areas of high and low deprivation, our comparative analysis revealed the strongest difference in technology-based practices was between the younger and older age groups (16-19 and 20-24 years). Almost every 20-24 year old reported having Internet access on his mobile phone and often used a more technical language during their discussions, mentioning IP addresses, firewalls, torrents (a computer file that contains metadata about files and folders to be distributed), which was something the younger respondents did not mention. This perhaps suggests greater use and familiarity with these technologies, and therefore integration in their lives, among the older men.

I’m doing alright. I’ve now got ten things in my house connected to the Internet. I’ve got one more thing, I’ve got a tablet now...two computers, X-Box, PlayStation, two phones, tablet, PSP [PlayStation Portable], Kindle…

Focus Group 8, 20-24, non-deprived

We identified three themes related to barriers and facilitators to successful implementation of an Internet-based screening program from the young men’s discussions: acceptability of proactive screening, confidentiality and privacy concerns, and language, style, and content.

### Acceptability of Proactive Screening

Overall, almost all of the young men considered an Internet-based screening approach to be acceptable and suggested they would use it, if it were offered to them. Participants described feeling inclined to be screened using this approach due to their perceptions of the ease and convenience with which they could be tested. In particular, they could avoid visiting a clinic and/or taking time off from work or education to do so.

The anonymous part of this is just brilliant compared to having to sit [at a clinic].

Male 1, Focus Group 7, 20-24, non-deprived

If I had to do it, if I was going to be, see myself round and I needed to get tested, I would choose this option [Internet screening] over going to the GP or the clinic.

Male 3, Focus Group 7, 20-24, non-deprived

However, these aspects were not valued by all, as a few teenage men in a group drawn from a deprived, semi-rural area thought clinic attendance would provide them with quicker access to a test.

I probably wouldn’t even use it [test kit]. I’d probably just get the bus up to [sexual health clinic] and let them give me a check-up.

Male 4, Focus Group 2, 16-19, deprived

No, I would just go to the clinic. I’d just rather do it than go online and need to wait. Just get it done there.

Male 5, Focus Group 2, 16-19, deprived

These men spoke of a nearby clinic offering convenience due to its location and their experience of attending gave them familiarity with the service, which they did not have for an online service. Nevertheless, these men spoke favorably of an Internet-based approach for “others”, which means that overall most men found the proposed approach to be acceptable.

### Privacy and Confidentiality Concerns

Participants, across almost all groups, described privacy and confidentiality concerns in relation to most aspects of the proposed Internet-based screening approach. Across these discussions, some men merely sought clarification on the confidentiality that would be offered and had no strong concerns about it. For example, some asked for clarification on the process of receiving their result, expressing a desire to choose the method they felt offered the greatest degree of confidentiality. However, others described possible scenarios of anxiety or conflict arising from being sent a screening invitation letter. One young man, who lived with his parents, envisaged conflict arising between him and his parents if a letter were to arrive for him:

If my ma finds it [letter] man, I’ll kill her before she kills me.

Male 4

Right, so is that an issue then, if your parents find out?

Facilitator 1

If my mum and dad found out, man, they’d kick me out again.

Male 4, Focus Group 1, 16-19, deprived

Another young man was concerned about his girlfriend assuming the invitation suggested unfaithfulness.

### Language, Style, and Content

Participants wanted screening invitation letters and a screening website to have content that is salient, credible, and straightforward. A degree of personalization was favored, particularly for the letters to be clearly addressed to individuals, so that letters would not be misinterpreted as “junk mail” and discarded without reading. The idea of screening “speaking to them” (of being personally relevant) emerged strongly across discussions. Indeed, some of the most excitable, animated, and enthusiastic discussions across the groups occurred when men were shown and allowed to browse five sample chlamydia screening websites on a laptop placed in front of them. Most discussions centered upon men’s design and content preferences; the level of detail almost every man provided about font size, color, images, and text was extremely rich. Participants were encouraged to reach a consensus within their groups on their broad design and style preferences, which aggregated across the groups included: careful use of imagery to appeal to a broad spectrum of men; no use of “text speak” (eg, RU clear, test 4U); use of an official health organization logo and colors (eg, NHS); minimal use of informal, chatty, humorous language; simple, straightforward text, presented using minimal length paragraphs; and the use of a “hide” button for privacy (enables the Web page to instantly change to the Google search page or minimizes the window).

Some men voiced uncertainty as to whether websites should be “cool” or serious. The following extract from a group discussion illustrates this, with two men attempting to convince a third that chlamydia screening websites should look “serious”:

See, the first one [website], I would not type my details.

Male 2

It’s a graffiti font there. I can’t take that seriously.

Male 1

Is that how bad websites look like, then?

Male 3

It’s not about being bad websites, but serving a purpose. In this case, it’s about health, it’s not about being cool, which that website aims…

Male 1

What’s wrong with being cool?

Male 3

I think it doesn’t tie in…

Male 1

So that people have actually…?

Male 3

Because I think it has to be straight to the point and professional, instead of being cool.

Male 1

I think if you’re actually thinking about saying what it says, that you’re concerned and you’re worried about it, so you’re not…you’re more about getting straight to business, not like funky websites

Male 2

Exactly. If you are worried about this, I think a funky website would be the last thing I would enjoy. So, what you have is chlamydia and they show you pictures of rabbits.

Male 1, Focus Group 12, 20-24, deprived

It was clear that images and use of color invoked more reaction than the text-based content, except for key headings or “tag lines” such as “RU Clear?”. It appeared that men felt patronized by the use of certain images and “text speak”. Men were keen to feel that there was an “authentic” voice in order to avoid discouraging them from engaging in screening—that authentic voice should be from youths rather than adults clearly masquerading as youth. Some men, particularly older and those from non-deprived areas, described STI testing as an adult issue, which led them to believe that the letters and websites should treat men in an adult fashion. Most thought the whole look and feel should be aimed at men around age 25 years to avoid being patronizing.

Why do they keep putting, like, “R U” and stuff? I actually don’t know anyone who texts like that anymore.

Male 2

Just overly, excessively cheesy [corny, lacking in taste].

Male 1, Focus Group 5, 16-19, non-deprived

 

I would feel quite patronized by that [website 2].

Male 2

Yeah, absolutely.

Male 5

You know, I don’t think, like, chlamydia testing should try to make itself cool, really, it’s not…

Male 4

I’d say the “R U clear” thing’s a bit unnecessary. It’s like, it’s different if you’re seven years old or something, but I think people our age can pick up so quickly or easily, if people are trying to look or an organization is trying to tap into a youth [sic].

Male 3, Focus Group 6, 16-19, non-deprived

However, most participants acknowledged the difficulty in designing a website that would appeal to all men aged 16-24.

## Discussion

### Principal Findings

In this paper, we analyzed data from 15 focus groups with young men aged 16-24 years (n=60) and drew over half the groups from areas of high deprivation. We identified a number of potential barriers and facilitators to Internet-based chlamydia screening, including: the acceptability of Internet-based screening to the target group, men’s confidentiality and privacy concerns, and language, style, and content across screening materials, including an invitation letter and a website. Our data illustrate the importance of not only taking time to develop messages that are framed appropriately for the target population, but to engage the target population in the design stage. We found levels of disconnect from technology, such as the Internet, among the younger men and men from deprived areas, which suggests that an Internet-based approach to STI screening (whether chlamydia-focused or broadened to include other STIs) may have the potential to widen inequalities in the absence of available, alternative screening opportunities.

### Strengths and Limitations

We presented sample letters and websites to men with a “real-world” screening context, which means they stated preferences and intentions, rather than actual behaviors. Intentions do not always predict behavior, as shown in a study of acceptance of herpes testing in adolescents and young adults [38], where many young people with high intentions of participating did not have a test. Thus, the high level of acceptability among the men in this study would not necessarily translate into high uptake of Internet-based screening. The focus group, by definition and design, allowed participants to be influenced by the group interaction [39]. Nevertheless, it is also a strength of the method for this work because sexual behaviors and attitudes are often related to peer influence and focus groups can therefore illuminate these shared meanings. Other limitations include the use of a female researcher, which may have influenced the performative aspects of the men’s participation and thereby influenced the data. *What* men chose to report and to *whom* has been discussed by others, who report differences in men’s stories according to the gender, ethnicity, or class of the interviewer [40]. Furthermore, our findings are based on a small number of men from a limited range of localities in Scotland and caution should be taken in any attempt to generalize from the results. Qualitative research provides rich descriptions of the particular, thus caution is always warranted for any attempt to generalize. We recommend further mixed methods research is carried out in order to enhance this work.

Our study focused on men, which necessarily means we offer no comparative analysis with women’s views. Previous work has described men as a hard-to-reach group and found many more women than men providing their views toward the development of a general sexual health website [33]. Our study prioritized men’s views and fills an important gap in the current literature. Our deliberate recruitment of men from areas of high deprivation is also a strength of this work, as it has enabled comparison of views by deprivation as well as age group, thereby not treating young men as a homogenous group.

### Comparison With Prior Work

Our findings point to the importance of message framing for how men may respond to the provision of a sexual health service, which aligns with recent work in different cultural and policy contexts [33,41-43]. Focus groups with American youth, which included young people in the development of technology-based interventions for sexual health promotion or service delivery, found issues such as the “authenticity of voice” were key to ensuring effective communication with youth [44]. Young people interviewed in a Canadian study by Davis et al (2012) “perceived the youthful messaging style as feigned” and that the level of seriousness absent from the reviewed websites rendered messages as being without value [41]; participants valued websites that had a professional and relatively serious tone. The young people who assisted in the development of the UK-based Sexunzipped website also sought websites they considered trustworthy and mature [33]. In our study, most men dismissed informal, casual styles and content as inappropriate for the seriousness of the issue of chlamydia infection.

Men in our study queried the extent of privacy and confidentiality offered by the screening approach we described, in relation to almost every aspect of the process. Clearly, these issues were of great importance and would be either barriers or facilitators to screening uptake, depending on how they are dealt with by intervention developers and then perceived by men. Apprehensiveness about who will know they sought screening (eg, from letters arriving at their parental home or a test kit arriving in the mail), how people may react to the knowledge they sought screening (eg, girlfriends believing a test suggests unfaithfulness), and how private their results would remain were of great concern to the men in our study. Clearly, their decision to participate in the screening we proposed would be dependent on them feeling confident that they were being offered the most private and confidential approach possible. The issue of privacy is one that has emerged across various contexts, such as with other Scottish youth [13], as well as in England [45], the United States [46,47], and Canada [48].

There are few qualitative studies that seek to obtain the views of youth toward the offer of chlamydia screening and even fewer that focus on the views of young men with which to compare. In our study, men from more deprived areas expressed different views toward technology use and Internet-based chlamydia screening than men from more affluent areas, reporting a lack of interest in engaging with particular technologies. Younger men and those from lower socioeconomic demographics who participated in Internet-based screening in the Netherlands were better reached with reminders by text message than email [49], although, overall, men participated less than women [25]. In contrast, a randomized controlled trial in Australia of the effects of text and email on young people’s sexual health had limited impact on men [50]. However, a text-based approach to sexual health promotion with San Francisco youth reached youth from low income backgrounds [44] and others have found low income minority groups can be reached in large proportions using the Internet [51]. Thus, there is emerging evidence, albeit variable, that these technologies could serve men better than “traditional” settings-based approaches, perhaps due to the way technology can deliver information and services. Our work aligns with the view that young people use technologies “that are dominant in their lives [and are a] fit with their own habitus, which, in turn, links to their social background” [52].

### Meaning of the Study

Our findings point to opportunities to refine the design and content of Internet-based sexual health interventions. For example, it is important that young men perceive a screening approach as offering privacy and confidentiality. This means intervention developers should seek the active involvement of men in the design stages, particularly younger men and those from areas of high deprivation—subgroups of men who have to date largely not been reached to the same level as other groups.

Our ability to engage men in this study should direct others to cease simply labelling them as “hard-to-reach”. Although there is a paucity of qualitative studies focusing on young men, work does show that men query the relevance of chlamydia screening to them believing it to be a “woman’s disease” [45,53], hold lower knowledge about chlamydia than women [54,55], and fear a swab-based test [56,57]. This underscores the importance of engaging various groups of men in the development of interventions to ensure that they, along with women and more affluent youth, shape future interventions. One man in this study, once the focus group ended, reflected: “We have been talking about chlamydia for the last hour, I think that’s cool…” [Focus Group 12, 20-24, deprived). Many men may wish to become part of a solution to rising chlamydia rates rather than be labelled as part of the problem.

### Conclusions

The findings of this study provide timely and important data on the benefits and challenges of Internet-based STI screening, within a context of no national screening program, poor engagement with men in screening, and challenges in engaging men from deprived areas. Although we have shown that an Internet-based approach has potential and technology is increasingly becoming integral to people’s lives, clearly some men engage with it more than others. Technology opens up opportunities for rethinking how we deliver sexual health services to young people, but we should be careful not to widen inequalities; we should consider carefully how to engage various groups of men and ensure we do not promote “one-size-fits-all” screening approaches.

## Multimedia Appendix 1

Websites shown to young men during focus group discussions.

## Abbreviations

Apps: applications
ECDC: European Centre for Disease Prevention Control
GP: general practitioner
ICT: information and communication technology
NCSP: National Chlamydia Screening Programme
SIMD: Scottish Index of Multiple Deprivation
SMS: short messaging services
STI: sexually transmitted infection
