# Supplementary figures and images for: Young Men’s Views Toward the Barriers and Facilitators of Internet-Based Chlamydia Trachomatis Screening: Qualitative Study
Source: J Med Internet Res. 2013 Dec 3;15(12):e265. doi: 10.2196/jmir.2628 (PMC3868974; doi:10.2196/jmir.2628)

## Slide 1
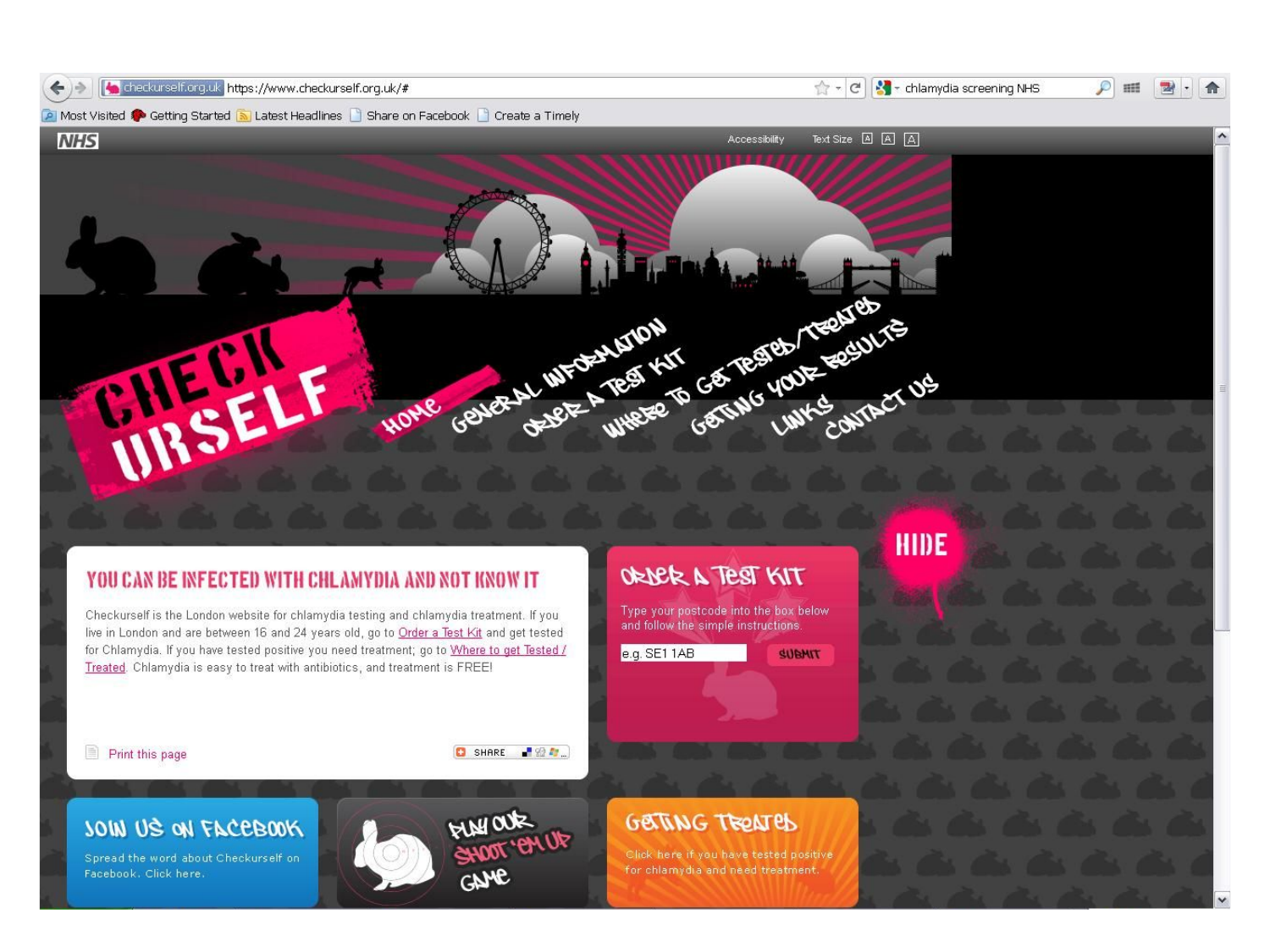

## Slide 2
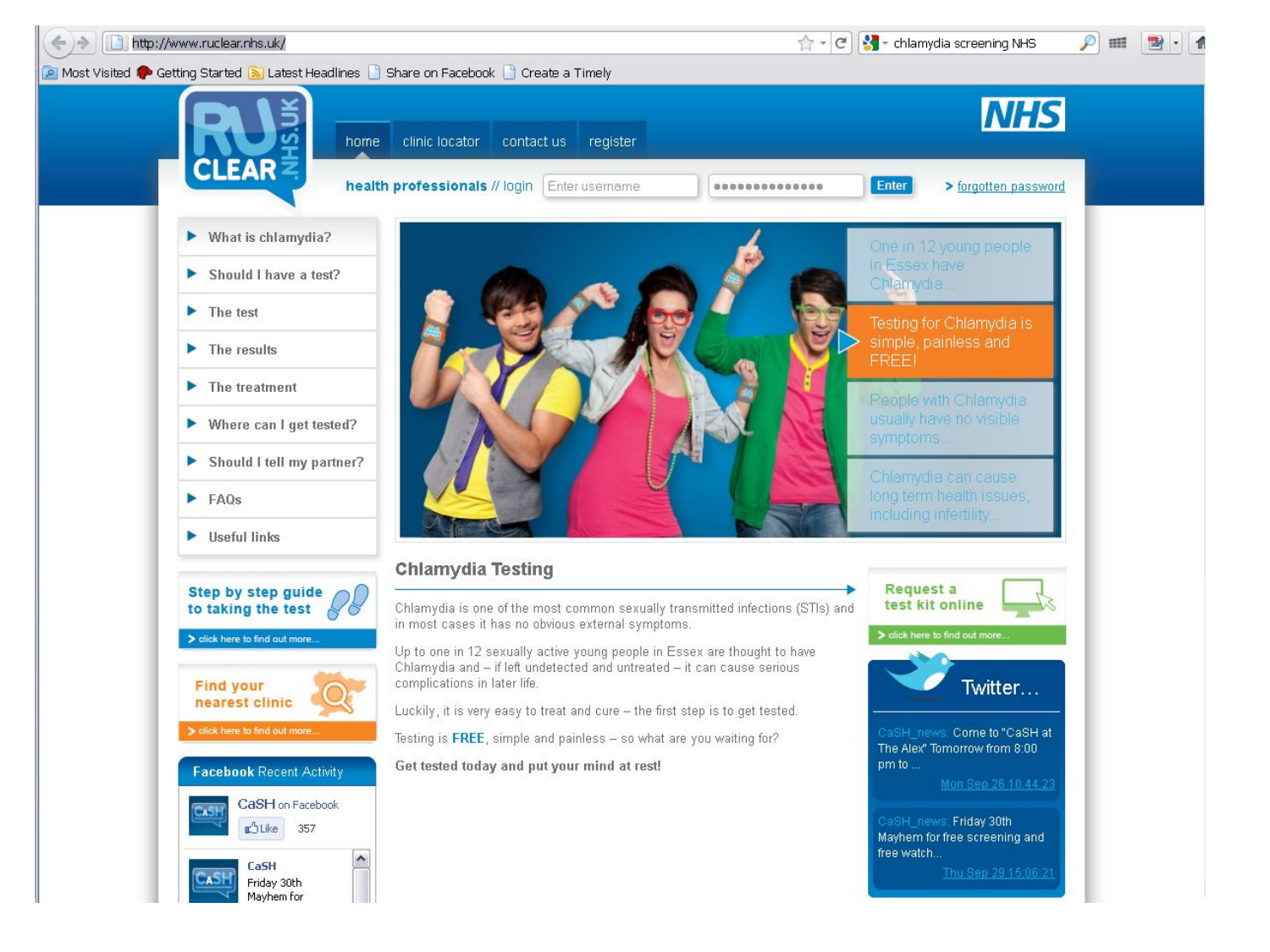

## Slide 3
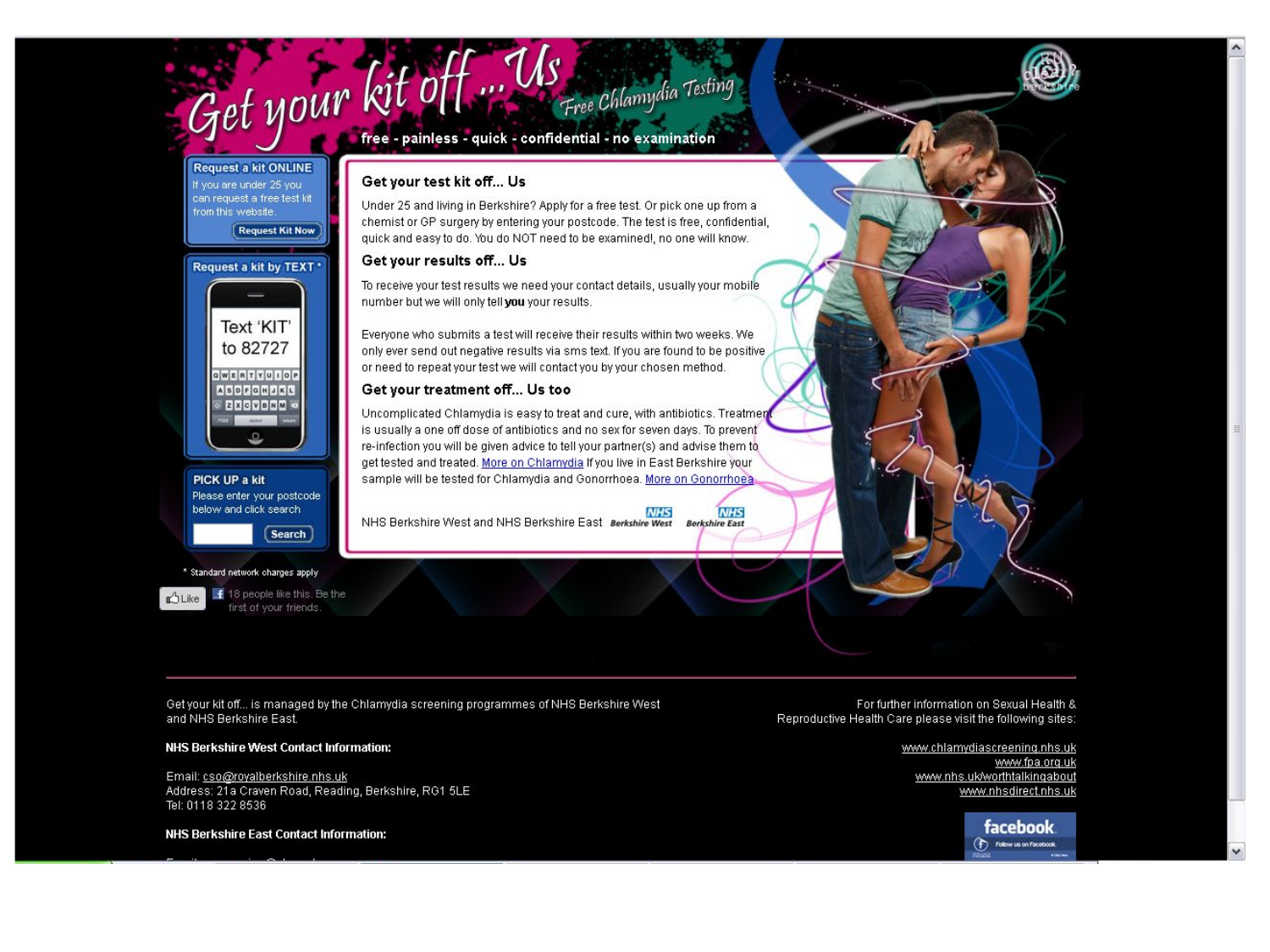

## Slide 4
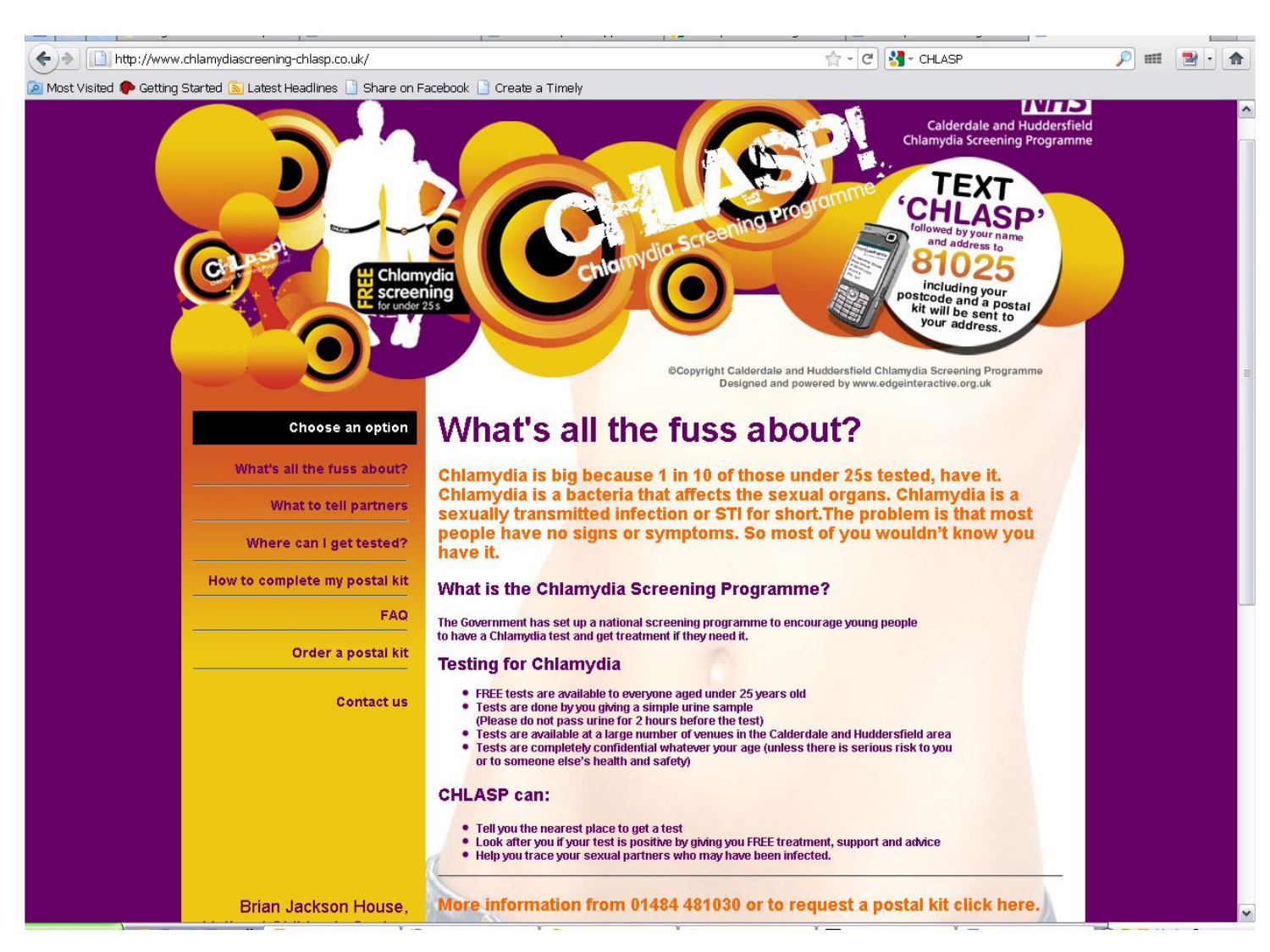

## Slide 5
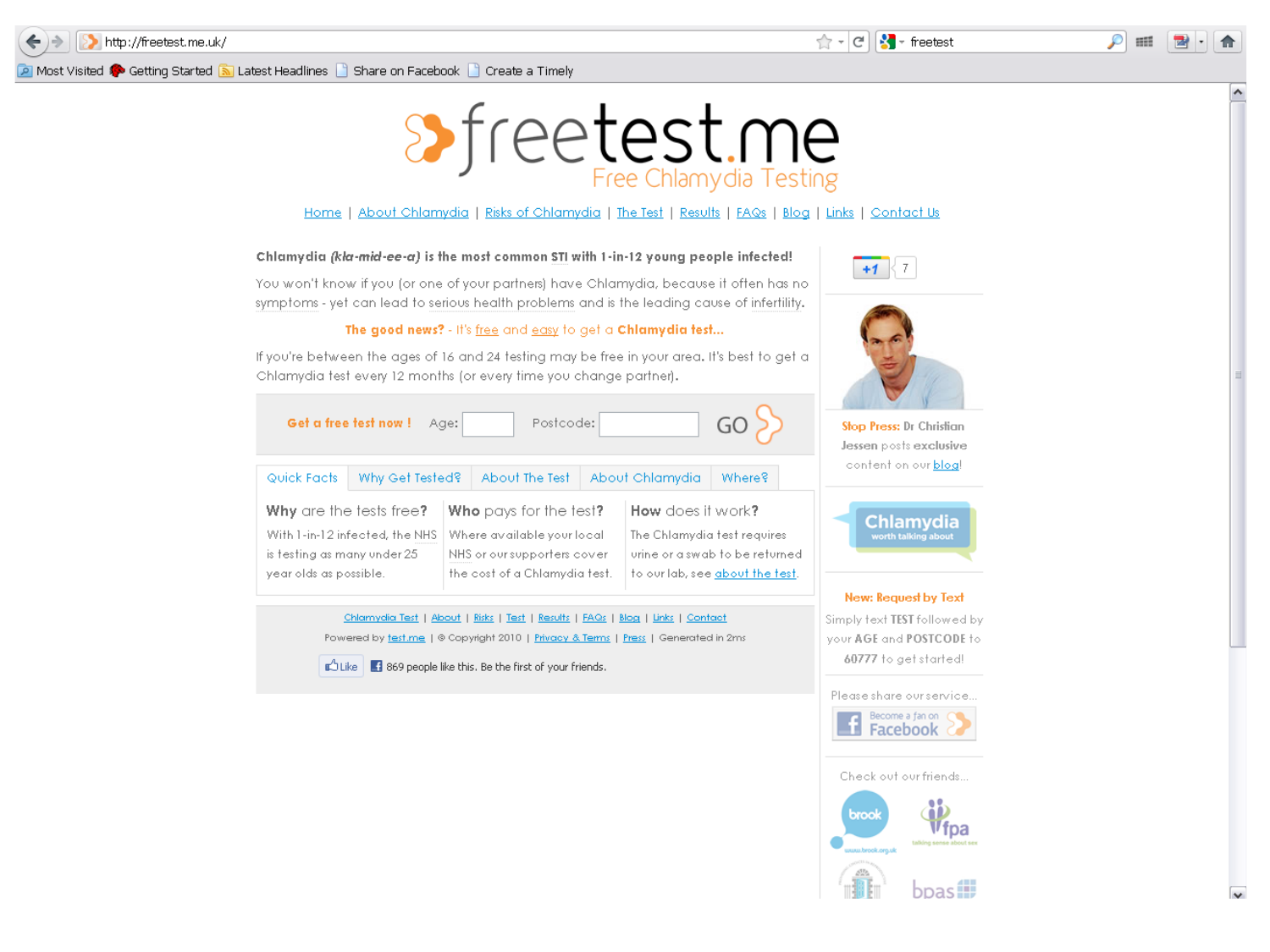

Supplement: Supplementary file 1 [file jmir_v15i12e265_app1.pptx]
